# Olive Industry Solid Waste-Based Biosorbent: Synthesis and Application in Wastewater Purification

**DOI:** 10.3390/polym15040797

**Published:** 2023-02-04

**Authors:** Angham Salahat, Othman Hamed, Abdalhadi Deghles, Khalil Azzaoui, Hisham Qrareya, Mohyeddin Assali, Waseem Mansour, Shehdeh Jodeh, Gül Gülenay Hacıosmanoğlu, Zehra Semra Can, Belkheir Hammouti, Asep Bayu Dani Nandiyanto, Alicia Ayerdi-Gotor, Larbi Rhazi

**Affiliations:** 1 Chemistry Department, Faculty of Science, An-Najah National University, Nablus P.O. Box 7, Palestine; 2 Scientific Research Department, Istiqlala University, Jericho P.O. Box 10, Palestine; 3 Laboratory of Engineering, Electrochemistry, Modeling and Environment, Faculty of Sciences, Sidi Mohamed Ben Abdellah University, Fez 30000, Morocco; 4 Industrial Chemistry Department, Arab American University, Jenin P.O Box 240, Palestine; 5 Department of Pharmacy, An-Najah National University, Nablus P.O. Box 7, Palestine; 6 Environmental Engineering Department, Marmara University, Istanbul 34840, Turkey; 7 Laboratory of Applied Chemistry and Environment LCAE, Faculty of Sciences, First Mohammed University, Oujda 60000, Morocco; 8 Departement Kimia, Universitas Pendidikan Indonesia, Bandung 40154, Indonesia; 9 Institut Polytechnique UniLaSalle, 19 Rue Pierre Waguet, BP 30313, 60026 Beauvais, France; 10 Institut Polytechnique UniLaSalle, Université d’Artois, ULR 7519, 19 Rue Pierre Waguet, BP 30313, 60026 Beauvais, France

**Keywords:** 3D polymer, cellulose, hemicellulose, lignin, Langmuir isotherm, wastewater, adsorption, kinetic

## Abstract

In this work, we present a process for converting olive industry solid waste (OISW) into a value-added material with ionic receptors for use in the removal of toxic metal ions from wastewater. This 3D polymer is a promising adsorbent for large-scale application, since it is a low-cost material made from agricultural waste and showed exceptional performance. The synthesis of the network polymer involved the carboxymethylation of OISW and curing of the carboxymethylated OISW at an elevated temperature to promote the formation of ester linkages between OISW’s components. FT-IR, atomic force microscopy, and thermal analysis were performed on the crosslinked product. The adsorption efficiency of the crosslinked carboxymethylated OISW toward Pb(II), Cu(II), and other toxic metal ions present in sewage was evaluated as a function of adsorbent dose, temperature, pH, time, and initial metal ion. The percentage removal of about 20 metal ions present in a sewage sample collected from a sewer plant located in the Palestinian Territories was determined. The adsorption efficiency did not drop even after six cycles of use. The kinetic study showed that the adsorption process follows the Langmuir isotherm model and the second-order adsorption rate. The experimental Qe values of 13.91 and 13.71 mg/g were obtained for Pb(II) and Cu(II) removal, respectively. The thermodynamic results confirm the spontaneous metal bonding to the receptor sites of the crosslinked carboxymethylated OISW.

## 1. Introduction

One of the most economically significant agri-food sectors in the Mediterranean and Middle Eastern regions is the olive oil industry. Due to the extent and scope of olive production worldwide, enormous amounts of unutilized agronomic waste are produced annually [1], creating serious environmental issues in the area.

The waste generated from the mills of the olive industry is made up of 43.8% solid waste and 56.2% liquid waste (OILW) [1,2]. The biological oxygen demand (BOD) and chemical oxygen demand (COD) values of these wastes are extraordinarily high, and they also contain hazardous amounts of polyphenols [1]. Environmentalists are concerned about the waste materials because they pose a significant disposal issue for waste management, which is problematic for the olive mills [1]. While OILW is typically disposed of through the sewage system, which has an impact on water quality, olive industry solid waste (OISW) is typically burned or allowed to rot in some countries, releasing CO_2_ into the atmosphere. Therefore, one of the biggest issues facing entrepreneurs considering rising environmental regulations is waste. Additionally, by disposing of the effluent or selling it for a cheap price, the olive business suffers economic loss.

Utilizing and transforming these wastes into beneficial and affordable commercial products is a difficult task; several studies [3,4,5,6,7,8,9,10,11,12,13,14,15,16,17,18,19,20,21,22,23] have reported examples on converting olive industry solid waste into product with various applications. None of the studies were related to using OISW in wastewater purification from toxic metal ions or other toxic organic matters. Wastewater purification and recycling have recently received a lot of attention from scientists. Several techniques such as adsorption, neutralization, ion exchange, filtration, and precipitation are used to remove heavy metal ions from contaminated wastewater. Among these methods, the adsorption technique has received the most attention [24,25,26,27,28,29,30].

The adsorption approach was chosen for this study as it is practical and affordable. There are numerous safe, recyclable, and environmentally friendly adsorbents that are reported in the literature [31,32,33]. Many other eco-friendly adsorbents with high efficiency for hazardous heavy metal ions prevalent in wastewater [17] are known in the literature. These adsorbents include those produced from neutral polymeric materials such as cellulose, lignin, and chitosan [34,35,36,37]. Although adsorbent technology has advanced quickly, those derived from the natural macromolecules mentioned above have not yet been completely investigated [38,39,40,41,42,43,44]. Cellulose nanocrystalline (CNC)-based adsorbents were among the most promising, but they had several issues with crystallinity that restrict the accessibility of backbone binding sites [45,46].

In this study, we developed a novel approach for combining the components of OISW into a 3D polymeric network with ionic sites for metal extraction from wastewater. The components of OISW include cellulose, hemicellulose, and lignin. They were carboxymethylated by reacting them with sodium chloroacetate in alkaline medium and then cured at a high temperature to promote the formation of ester linkages between the components and the formation of the target 3D network polymer.

## 2. Materials and Methods

### 2.1. Material

Deionized water was used in this work, collected using a 18.2 MΩ cm Millipore (Millipore Corporation, Burlington, MA, USA). All chemicals and reagents used in this work were of analytical grade and were purchased from Sigma-Aldrich chemical company (Jerusalem, Israel) and used as received. The chemicals included sodium chloroacetate, copper(II) sulfate pentahydrate, and lead(II) nitrate. All solutions were prepared using deionized water. OISW used in this work was collected from an olive mill located in the city of Tukaram (Palestinian Territories).

### 2.2. Methods

#### 2.2.1. Characterization

The thermal behaviors of the CMOISW and curing product were evaluated using the thermo-gravimetric analysis TG/DSC Star System (Mettler-Toledo, Columbus, OH, USA), heated at a rate of 5 °C/min from 25.0 to 1100.0 °C. FT-IR spectra were recorded on a Fourier transform infrared (FT-IR) spectrometer (Nicolet 6700, Thermo Fisher Scientific, Waltham, MA, USA). The FT-IR resolution was set at 4 cm^−1^. The spectra were recorded in the range of 400–4000 cm^−1^ with 128 scans. For controlled data analysis, Mettler-Toledo STARE software version 10.0 was employed.

The concentrations of control ions Pb(II) and Cu(II) were determined using a flame atomic absorption spectrometer (FAAS, ICE3500 AA System, Thermo Scientific, Horsham, UK). The quantitative and qualitative examinations of wastewater were performed using inductively coupled plasma mass spectrometry (ICP-MS, CAPTM RQ ICP-MS, Thermo Fisher Scientific, Waltham, MA, USA). The mean of three runs was provided for each analysis. The pH values of the aqueous solutions were determined using the pH electrode by Mettler-Toledo (Mettler-Toledo (Schweiz) GmbH).

#### 2.2.2. Purification of Olive Industry Solid Waste

A 100.00 g sample of dried OISW was ground by passing it through a Wiley mill fitted with a 200-micron screen (Thomas Wiley^®^ Mini Cutting Mills, Swedesboro, NJ, USA). The ground OISW was stirred in acidic solution with a pH of 3.0 (0.50 L) for about 60 min to remove water-soluble extractives. The solid was then collected by suction filtration and washed several times with water until the filtrate was neutral and clear. The collected OIWS was allowed to dry at room temperature.

#### 2.2.3. Soxhlet Extraction

Soxhlet extraction is one of the most popular techniques for the extraction of organic impurities from a solid material. In this work, it was performed on OISW to remove residual olive oil and other organic extractives. The OISW collected from the previous experiment was placed in the extractor and extracted with ethyl acetate (500 mL) for 2.0 h. The collected OISW sample was dried at room temperature and stored in a refrigerator for further reactions.

#### 2.2.4. Carboxymethylation of OISW (CMOISW)

A 10.00 g sample of purified OISW was placed in a three-neck round bottom flask fitted with a large magnetic bar, a thermometer, and a condenser attached to a bubbler. To the OISW in the flask, 150.00 mL of isopropyl alcohol and 15.00 mL of distilled water were added. Then, 12.00 mL of 50.00% NaOH solution was added to the mixture dropwise with a syringe over a period of 10.0 min. After that, 21.00 g (0.18 mol) of sodium chloroacetate was added in one portion. The flask contents were heated to 62 °C in 30.0 min and kept at this temperature for 60 min. The reaction was then quenched by the addition of 8.0 mL of acetic acid. The produced solid was collected by suction filtration and washed three times with a methanol/water solution (2:1 ratio by volume). The last wash was performed with only acetone. The mass of the produced carboxymethylated OISW polymer was 12.00 g.

#### 2.2.5. Thermal Curing

A sample of CMOISW (1.00 g) was thermally treated at 160 °C for 20.00 min. The produced sample was collected and evaluated for water solubility and carboxyl content as shown below.

#### 2.2.6. Carboxyl Content of CMOISW

The degree of substitution (DS) of CMOISW was determined according to the standard procedure ASTM 2005 [47]. A sample of ground CMOISW (5.0 g) was suspended in 95% ethyl alcohol (100.0 mL) and stirred for 10 min. Then, 5.0 mL of concentrated nitric acid was added to the suspension. The mixture was agitated for 30 min at room temperature. The solvent was decanted and the solid residue was washed with 60% ethanol (5 × 50 mL). Each time, the solid was stirred in the ethanol solution for 20 min, then decanted. The last wash was performed with methanol. The precipitate was then collected by suction filtration and dried under vacuum at 60 °C for 2 h to ensure complete removal of solvent. A 1.0 g sample of dried CMOISW was suspended in distilled water (100 mL) and 25.0 mL of 0.3N NaOH. The mixture was agitated for 30 min. The produced clear solution was titrated with 0.3N HCI in the presence of a phenolphthalein indicator. The end point was reached when the pink color converted to clear. The degree of substitution was calculated according to Equations (15) and (16).

#### 2.2.7. Water Solubility of CMOISW

A 1.00 g sample of CMOISW was drenched in water (25.00 mL) and agitated for about 2 h, collected by filtration, and dried in an oven adjusted at 110 °C for one hour. The sample was cooled in a desiccator and weighed. A 0.04 g reduction in the sample mass was recorded.

#### 2.2.8. Wastewater Purification

A sewage sample collected from one of the wastewater purification plants in Palestine was used in this study. The type and percentage of metal ions present in the sample were analyzed by the Water Center, An-Najah National University, Palestine, using ICP-AES. Two 20 mL vials each containing a 10 mL sample of wastewater were prepared. One of them was treated with 50 mg of the adsorbent CMOISW and the second was kept as a reference. The pH of both solutions was adjusted to 6.5. The vials’ contents were shaken at room temperature for 30 min using a thermostat equipped with a shaker. A 5.0 mL sample of each mixture was withdrawn and filtered through a 0.45 µm syringe filter and analyzed by ICP-AES for residual metal ion concentrations.

#### 2.2.9. Adsorption

Glass vials (20.0 mL) were used for the adsorption operations; they were clamped in a water bath with a thermostat and mixed for the determined time. Copper (II) and lead (II) ions were chosen as model metal ions in this work. The adsorption process was carried out using the batch approach [37,38,39]. Adsorption was carried out at 25 °C using various amounts of adsorbent and solutions of metal ions with concentrations ranging from 10 to 50 mg/L. The percentage of metal ions removed was studied in relation to a number of parameters, including adsorbent dose, initial metal ion concentration (*C*_0_, ppm), mixing duration (t, min), temperature (T, °C), and pH value; the pH was altered by adding either HNO_3_ or NaOH. A plastic syringe (10.0 mL) fitted with a filter (0.45 µm) was used to obtain filtered samples for analysis. The change in the metal ion content was monitored by flame atomic absorption spectroscopy at a wavelength of 217.0 nm. Equations (1) and (2) were used to calculate the adsorption capacity of the cured CMOISW.

Thermodynamic parameters were used to assess the nature of the adsorption process [38,39,40,41].

(1) $$\%\mathrm{Removal}=\frac{C_{0}-C_{e}}{C_{0}}\times 100$$

(2) $$Q_{e}=\frac{C_{0}-C_{e}}{W}V$$

where *C*_0_ and *C_e_* are the starting metal ion and the equilibrium metal ion concentration in ppm, respectively. *Q_e_* is the equilibrium adsorption capacity in ppm, *W* is the weight in mg of the absorbent, and *V* is the volume in *L* of the solution [42].

#### 2.2.10. Isotherm

The Langmuir isotherm model is presented in Equations (3) and (4):

(3) $$\frac{\;C_{e}}{Q_{e}}=\frac{1}{q_{max}}C_{e}+\frac{1}{q_{max}K_{L}}$$

where *C_e_* is the concentration of metal ion in ppm and *Q_e_* is the amount of metal ion extracted per unit mass of CMOISW at equilibrium (mg/g), *q_max_* is the highest single layer adsorption capacity of the polymer (mg/g), and *K_L_* (L/mg) represents the Langmuir constant [48,49,50].

The Langmuir isotherm model can be used to forecast whether adsorption will be favorable or unfavorable using the dimensionless constant separation factor provided in Equation (4).

(4) $$\;R_{L}=\frac{1}{1+K_{L\;}C_{e}}$$

where *C*_0_ stands for the initial metal ion concertation and *K_L_* stands for the Langmuir constant. If the *R_L_* value exceeds one, the adsorption is deemed to be unfavorable; it will be favorable or linear if it is between zero and one.

The Freundlich isotherm model shown in Equations (5) and (6) symbolizes the heterogeneous surface energy non-ideal adsorption process.

(5) $$\ln(Q_{e\;})=\ln k_{f}+\frac{1}{n}\ln C_{e}$$

*Q_e_* = *K_F_ C_e_*^1/n^ (6)

where 1/*n* is the adsorption intensity and *K_F_* stands for a constant that denotes the relative adsorption capacity [51,52].

#### 2.2.11. Adsorption Kinetics

The rate of metal ion adsorption on a CMOISW surface was examined using the pseudo-first-order and pseudo-second-order kinetic models shown below. The linearized versions of the rate equations were calculated using Equations (7)–(10) [52].

(7) $$\ln(q_{e\;}-q_{t})=\ln q_{e}-K_{1}\;t$$

(8) $$\frac{t}{q_{t}}=\frac{1}{K_{2}q_{e}^{2}}+\frac{t}{q_{e}}$$

*Q_t_* = *K*_id_t^1/2^ + Z (9)

(10) $$\ln\frac{K_{\left(T_{2\;}\right)}}{K_{\left(T_{1\;}\right)}}=\frac{Ea}{R}\cdot \left(\frac{1}{T_{1}}-\frac{1}{T_{2}}\right)$$

ln(1 − F) = −*K*_fd_ × *t* (11)

where *Q_t_* is the temperature-dependent adsorption capacity and *q_e_* is the equilibrium adsorption capacity (mg/g). *K*_1_ stands for the pseudo-first-order rate constant (min), and *K*_2_ for the pseudo-second-order rate constant (g/mg.min). Z (mg/g) can be used to determine the boundary layer thickness, where *K*_id_ is the diffusion rate constant measured in mg/g.min^1/2^. In Equation (11), *K*_fd_ (min^−1^) stands for the rate of film diffusion, and F represents the fractional accomplishment of equilibrium (F = *q_t_/q_e_*).

Equations (12)–(14) were used to compute the enthalpy (ΔH°), Gibbs energy (ΔG°), and entropy (ΔS°).

K_c_ = C_ads_/C_e_ (12)

ΔG° = −*RT*lnK_c_ (13)

(14) $$\ln K_{s}=\frac{∆S}{R}-\frac{∆\;H}{RT}$$

where *T* (K) is the solution temperature, *R* is the ideal gas constant (J/mol K), C_ads_ is the equilibrium Pb(II) adsorbed quantity (mg/L), C_e_ is the equilibrium concentration (mg/L) in the supernatant, and K_c_ is an apparent constant of the thermodynamics [53].

## 3. Results

**Figure 1:**
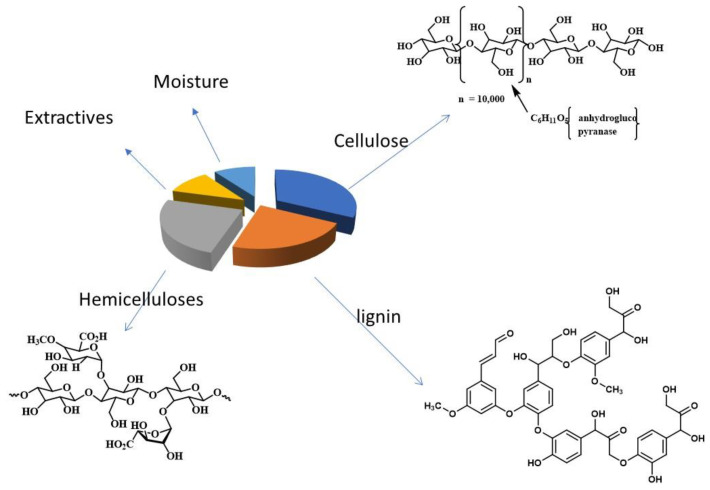
A diagram summarizing the components of OISW.

The solid waste of the olive industry is composed mainly of three components: cellulose, lignin, and hemicelluloses. The structures of these components are shown in Figure 1. The repeat units of the three components contain hydroxyl groups that are known to be a precursor for a variety of functional groups. Previous studies showed that cellulose is the major component of OISW [44,53].

### 3.1. Synthesis of Carboxymethylated OISW

**Figure 2:**
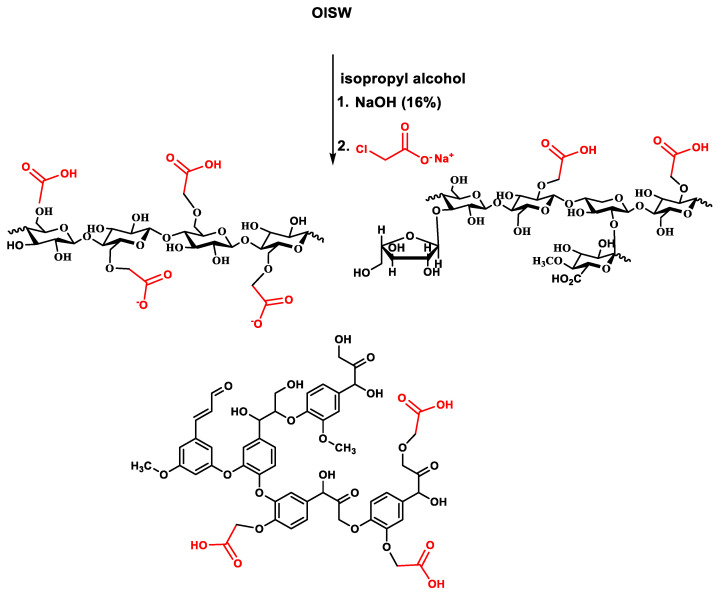
A summary of the carboxymethylation process of OISW.

A sample of OISW was purified from residual metal ions and organic extractives mainly composed of fatty acids, fatty alcohols, and fatty esters [44,53] by washing it with acid solution and Soxhlet extraction using ethyl acetate. The purified OISW was suspended in isopropyl alcohol and converted to alkoxide by reacting it with an aqueous solution of sodium hydroxide (50 wt %). The produced alkoxide then reacted with sodium chloroacetate at about 60 °C to produce carboxymethylated OISW (CMOISW). The reactants were mixed in a ratio that allowed the formation of partial carboxymethylated OISW, as shown in Figure 2. The carboxyl content was determined according to an ASTM 2005 method [47] based on cellulose molar mass (162.0 g/anhydrous glucose repeat unit), and 58 was the net increment in the molar mass of the anhydrous glucose repeat unit for every substituted COOH group. DS was determined to be about 0.72.

Carboxyl milliequivalents in CMOISW = (V_NaOH (mL)_N_NaOH_ − V_HCl(mL)_N_HCl_)/Mass (g) of CMOISW (15)

DS = 0.162 × Carboxyl milliequivalents/(1 − 0.058 × Carboxyl milliequivalents)(16)

**Figure 3:**
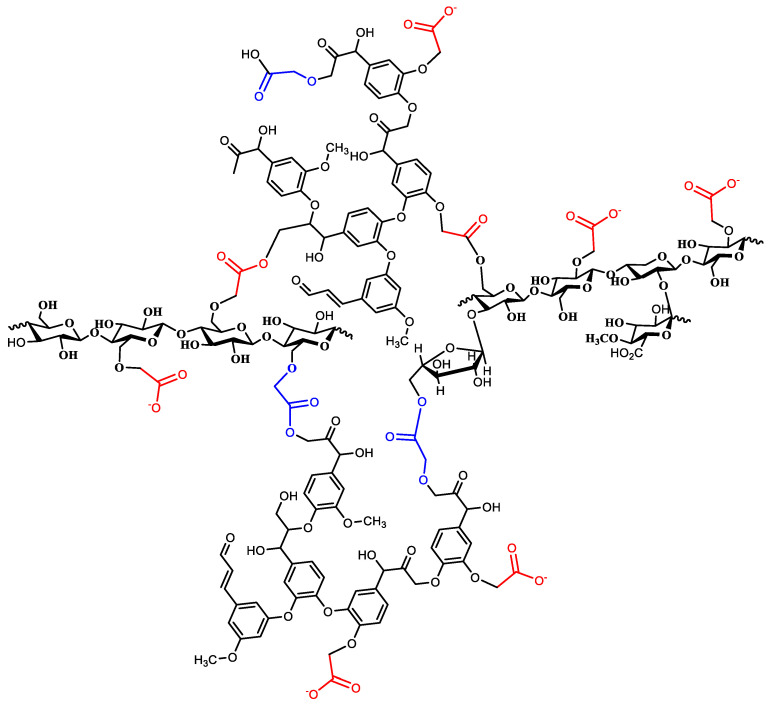
A representative figure of the cured (crosslinked) CMOISW.

The three carboxymethylated macromolecules were then heated at an elevated temperature to cause thermal curing. Each of the three macromolecules contained OH and carboxyl functional groups. Both groups tended to undergo a condensation reaction at an elevated temperature to form an ester linkage with the loss of water molecules. The result is that a 3D structure of covalently bonded lignin, cellulose, and hemicelluloses forms, as shown in Figure 3. The produced structure is loaded with chelating ligands for metal ions; in addition, it is expected to be highly porous.

**Figure 4:**
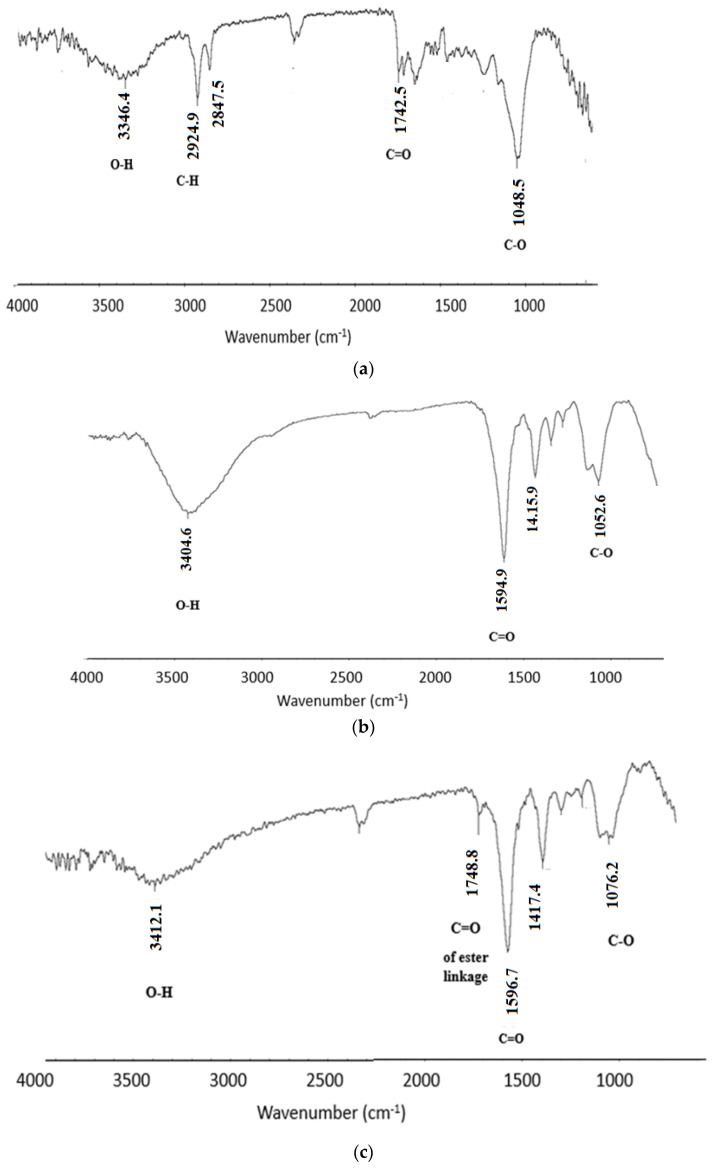
FTIR spectra of (**a**) OISW. (**b**) carboxymethylated OISW, and (**c**) thermally cured carboxymethylated OISW (CMOISW).

As mentioned earlier, the OISW is composed of cellulose, lignin, and hemicelluloses; the IR spectrum (Figure 4a) of the waste clearly shows peaks corresponding to the functional groups present in the OISW components. The spectrum shows a band at 3346 cm^−1^ corresponding to O-H stretching and two bands at 2924.9 and 2847.5 cm^−1^ corresponding to C-H stretching. The spectrum also shows several bands in the region of 1650 to 1742 that could be attributed to C=O of ester, aldehyde, and carboxyl functional groups, and several weak bands appear at about 1600–1610 corresponding to C=C of lignin aromatic rings, in addition to the O-C-O that mainly represents the glycosidic linkage in cellulose and hemicelluloses. The IR spectrum of the carboxymethylated OISW is shown in Figure 4b; the spectrum shows a broad band that stretches from 3500 to 2800 cm^−1^ corresponding to O-H of carboxyl and alcohol. A strong band appears at 1594 cm^−1^ corresponding to the stretching of C=O of the carboxylate functionality. The bands that represent the carbonyl of aldehyde and ester appear in the IR of OISW and disappear in the spectrum of CMOISW. This could be attributed to the treatment with NaOH during the carboxymethylation process, since under these conditions, the ester undergoes hydrolysis, and the aldehyde undergoes oxidation. The IR spectrum of CMOISW after curing shows a new band at 1748 cm^−1^ corresponding to the C=O of ester linkage that is formed by a condensation reaction between carboxyl and the O-H groups of the carboxymethylated OISW components.

**Figure 5:**
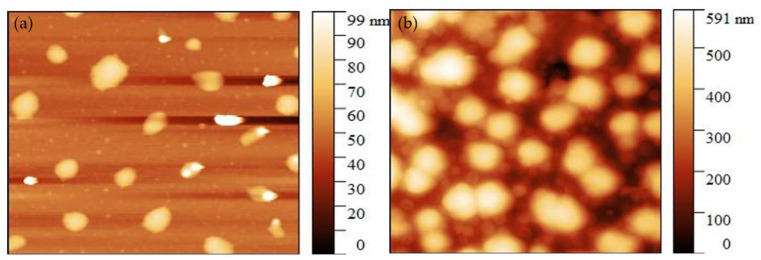
Atomic force microscope image of (**a**) CMOISW and (**b**) thermally cured OISW carboxymethylated OISW.

**Figure 6:**
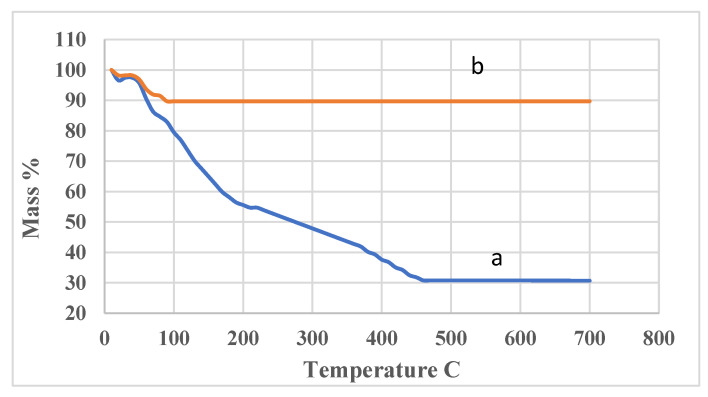
Thermal gravimetric analysis of (**a**) carboxymethylated OISW (**b**) thermal cured carboxymethylated OISW.

The images obtained by the atomic force microscope (AFM) of the CMOISW are shown in Figure 5 The average diameter for the CMOISW was about 30 *±* 2 nm. Figure 6b shows that for the thermally cured CMOISW, the particle size increased to 300 ± 15 nm. The increase in the particle dimensions could be related to the particles fusing during the curing process.

The thermal stability of CMOISW was measured by the TGA method. The obtained results are shown in Figure 6. CMOISW showed a major weight loss at 250 °C; the loss could be attributed to the decarboxylation and the fragmentation of cellulose and hemicelluloses. However, the cured CMOISW showed a minor weight loss at about 100 °C, which could be due to the evaporation of residual moisture present in the structure. The polymer showed very high thermal stability; heating it up to 800 °C showed a loss of only 10% of weight, and about 90% of its weight was reserved.

### 3.2. Adsorption Process

Batch adsorption experiments were conducted to test the efficacies of the thermally cured CMOISW for heavy metal adsorption, and to determine the optimum conditions for heavy metal removal from wastewater. Pb(II) and Cu(II) were selected as model ions in this study. The residual concentrations of Pb(II) and Cu(II) were determined by FAAS.

#### Effects of Various Parameters

##### Solution pH

**Figure 7:**
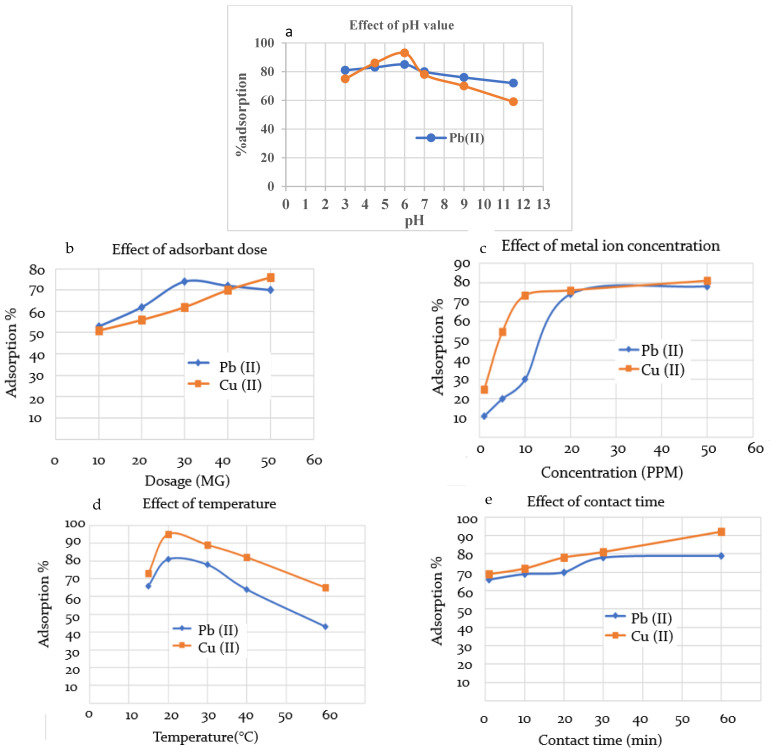
The effect of (**a**) pH on adsorption efficiency (time 30 min, temperature 25 °C, adsorbent dose 50.0 mg, metal ion concentration 30.0 mg, and solution volume 10.0 mL), (**b**) adsorbent dose (CMOISW) (time 30 min, temperature 25 °C. pH 6.0, metal ion concentration 30.0 mg, and solution volume 10.0 mL), (**c**) metal initial concentration (time 30 min, temperature 25 °C, adsorbent dose 50.0 mg, pH 6.0, and solution volume 10.0 mL), (**d**) temperature (time, 30 min, temperature 25 °C, adsorbent dose 50.0 mg, metal ion concentration 30.0 mg, and solution volume 10.0 mL), and (**e**) contact time (temperature 25 °C, adsorbent dose 50.0 mg, metal ion concentration 30.0 mg, and solution volume 10.0 mL).

The solution pH is an important factor that affects both adsorbent and adsorbate, since the ability of the functional groups to perform as a metal binder varies with pH value as they transfer from protonated to ionic, going from low pH to high. The effect of pH on the extraction ability of the cured CMOISW was evaluated while the other parameters were kept constant with an adsorbent dose of 50.0 mg, metal ion concentration of 30.0 mg, and solution volume of 10.0 mL. The adsorption was performed for 30.0 min at room temperature. The results are shown in Figure 7a. The results show that a significant removal of metal ions occurred at a pH of about 6.0. Since at this pH, the carboxyl group is present in the ionic state, the carboxyl group is converted to a strong one.

##### Adsorbent Dose

Various amounts of CMOISW adsorbent dosage (10.0 mg to 60.0 mg) were used in this extraction experiment; other parameters such as solution volume of metal ion, initial concertation of Pb(II) and Cu(II), and pH were kept constant at 10.0 mL, 20.0 ppm, and 6.0, respectively. Adsorption was performed at room temperature for 30.0 min. The obtained results showed that the optimum amount of dosage is about 30.0 and 50.0 mg for extracting Pb(II) and Cu(II), respectively (Figure 7b), with a maximum removal of about 74%.

##### Metal Ion Initial Concentration

The effect of the initial concentration of Pb(II) and Cu(II) on the efficiency of CMOISW was also studied while keeping the other parameters of solution volume, pH, time, temperature, and adsorbate dose constant at 10 mL, 6.5, 30 min, 25 °C, and 50.0 mg, respectively. The results are shown in Figure 7c. The maximum %removal was reached at 20.00 ppm of metal ion concentration. Increasing concentration did not show any effect on the %removal. As the initial concentration increased from 2.0 ppm to 20.0 ppm, the %removal increased and reached the maximum at 20.0 ppm. At a low concentration, there are plenty of receptors on the adsorbent surface, and the adsorption process is regulated by an ion diffusion mechanism [45,54]. The availability of the metal receptors begins to drop as the concertation increases due to saturation, which also results in the adsorption efficacy becoming constant.

##### Temperature

The percent removal of Pb(II)and Cu(II) as a function of temperature was studied in the range of 10 to 60 °C. The highest removal was obtained at room temperature, as shown in Figure 7d. However, the efficiency for CMOISW was dropped as the temperature increased to 60 °C, which indicates that the adsorption is exothermic and requires no or little heat. The kinetic energy of the adsorbed particles on the adsorbent surface increases as the temperature rises, and this increases the probability of their detachment from the adsorbent surface [55,56,57,58]. Moreover, the decline in adsorption at higher temperature can be related to the increase in the solubility of ions with temperature [56].

**Figure 8:**
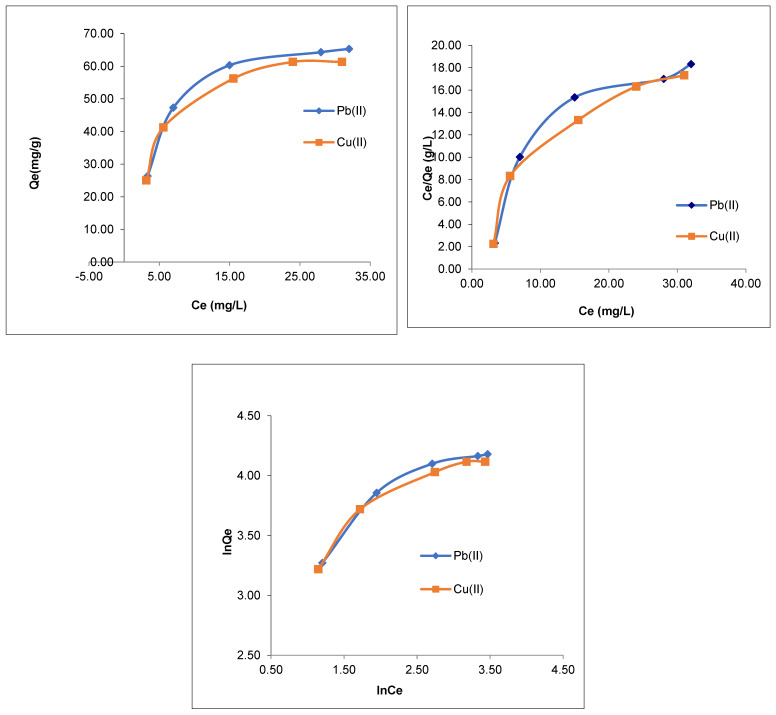
Langmuir and Freundlich adsorption plots of Pb(II) and Cu(II) ions on CMOISW.

The percent removal of Pb(II) and Cu(II) as a function of temperature was investigated. According to the obtained results shown in Figure 8d, the maximum adsorption was achieved at room temperature. However, as the temperature rose to 60 °C, the efficiency for CMOISW decreased, indicating that the adsorption is exothermic and does not require heat. As the temperature rises, the kinetic energy of the adsorbed particles on the adsorbent surface increases, increasing the likelihood that they will separate from the surface [51,52,53]. Additionally, the rise in ion solubility with temperature may be connected to the decrease in adsorption at higher temperatures [39,58].

##### Mixing Time

While the parameters of solution volume, pH, initial concentration of Pb(II), temperature, and adsorbent dose were held constant at 10 mL, 6.0, 20 ppm, 25 °C, and 20 mg, respectively, the influence of the mixing time on the adsorption effectiveness of CMOISW was examined. Figure 7e demonstrates that the percentages of Pb(II) and Cu(II) removal grew somewhat with time until becoming constant at 30 min (80% removal) for Pb(II), but it took longer for Cu(II) to achieve its maximum removal at about 60 min (90% removal). The availability of the adsorption receptors, which diminishes with time due to adsorption, might explain the results; by about 30 min, practically all metal receptors are occupied.

### 3.3. Wastewater Purification from Toxic Metal Ions

A sample of real wastewater was collected from a wastewater treatment plant located in Jericho (Palestine) and subjected to treatment with the adsorbate CMOISW using the optimum determined adsorption conditions. The initial concentrations in ppm of the metal ions and after adsorption experiments were determined using ICP-MS. A summary of the results is shown in Table 1. The CMOISW showed moderate to high removal efficiencies toward about 15 metal ions were achieved.

### 3.4. Adsorption Mechanism

#### Isotherm

The Langmuir (Equation (3)) and Freundlich isotherm (Equation (5)) models were followed to determine the adsorption equilibrium between the Pb(II) and Cu(II) ions and the 3D polymer CMOISW in water [38]. They were also utilized to assess the metal ion dispersion on the CMOISW surfaces at equilibrium. One of the factors that may affect the type of isotherm followed in the adsorption is the correlation coefficient (R^2^) [38].

Data listed in Table 2 represent the values of all adjustable parameters obtained from Figure 8. As shown in Table 2, the obtained data showed that the correlation coefficients obtained using the Freundlich isothermal model are lower, demonstrating that Pb and Cu cations adsorption adhere to the Langmuir equation, in which Pb(II) and Cu(II) cations are dispersed uniformly and evenly over the polymer porous surface. For various dosages of polymer adsorbent, the separation factor R_L_ ranges from 0 to less than 1 (Table 2). This demonstrates the affinity of CMOISW toward the relevant metal ions.

### 3.5. Kinetics of Pb(II) and Cu(II) Ion Adsorption

The Pb(II) and Cu(II) ion adsorption by CMOISW was analyzed using various kinetic models to understand the nature of the adsorption mechanism. Pseudo-first- and second-order models, two of the most popular kinetic models, were followed to simulate the metal adsorption by the CMOISW adsorbents. The used kinetic models are depicted in Equations (7) and (8) [39].

**Figure 9:**
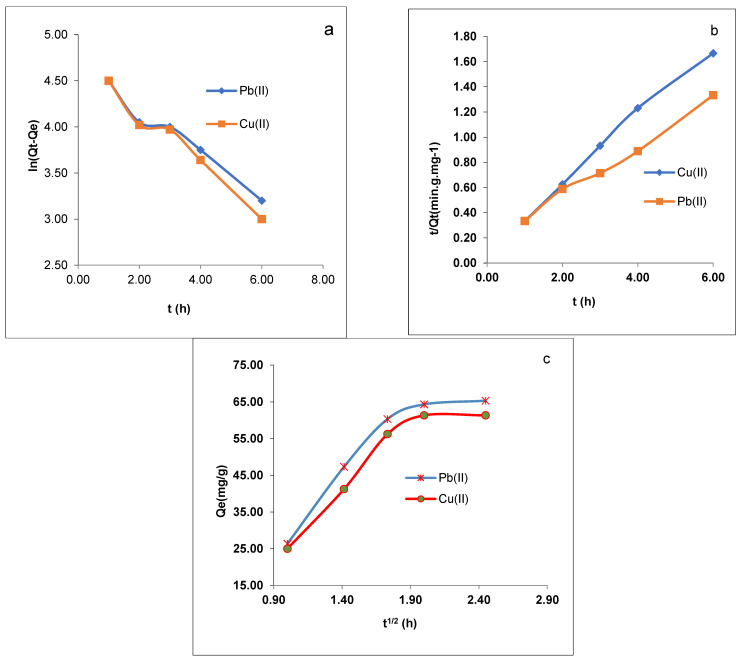
(**a**) Pseudo-first-order model, (**b**) pseudo-second-order model, and (**c**) intraparticle diffusion model for the Pb(II) and Cu(II) ion adsorption onto CMOISW at different concentrations.

The obtained equations’ parameter values are summarized in Table 3 and Figure 9. The value of K_1_ is provided by the plots of ln (q_e_–q_t_) vs. t (Figure 9a), while the values of K_2_ and the adsorption capacity q_e_ are provided by the slope and intercept of the plot of t/Q_t_ vs. t (Figure 9b), and K_id_ and Z are calculated by plotting Q_e_ vs. t^1/2^ (Figure 9c).

The results obtained from the experimental part reveal that the value of R^2^ for the pseudo-second-order kinetics model (0.9916 to 0.9933) is higher than that of the pseudo-first-order kinetics model (0.916 to 0.9035) of Pb (II) and Cu (II) ions onto CMOISW, respectively.

The values indicate that the Pb(II) and Cu (II) adsorption on the foam surfaces conforms to the pseudo-second-order model (Table 3 and Figure 9b).

K_id_ and Z were calculated from Figure 9c (Q_e_ vs. t^1/2^); their values are shown in Table 3. Several rate-limiting processes could be available for the adsorption process, since all the graphs shown in Figure 9 are straight lines and do not cross their respective origins.

We may infer from the initial graph’s linearity in Figure 9b that Pb(II) and Cu(II) adsorption on CMOISW starts with an instantaneous adsorption on the external surface, which results in a chemical complexation between metal ions and surface functional groups [38,39]. The other steps were also linear, showing that Pb(II) and Cu(II) ions were gradually adsorbing and that the rate of intraparticle diffusion was being constrained.

Table 3 demonstrates that while the outside mass transfer potential decreased and the top layer expanded, the inner mass transfer potential increased. Equation (9) was used to determine the energy of activation of the adsorption at 298 and 323 K.

These results help explain how temperature impacts the Pb(II) and Cu(II) ions’ ability to adsorb on CMOISW.

### 3.6. Thermodynamics study

To comprehend the spontaneity and the sort of adsorption, the values of the parameters of standard free energy, standard enthalpy, and standard entropy have been calculated.

**Figure 10:**
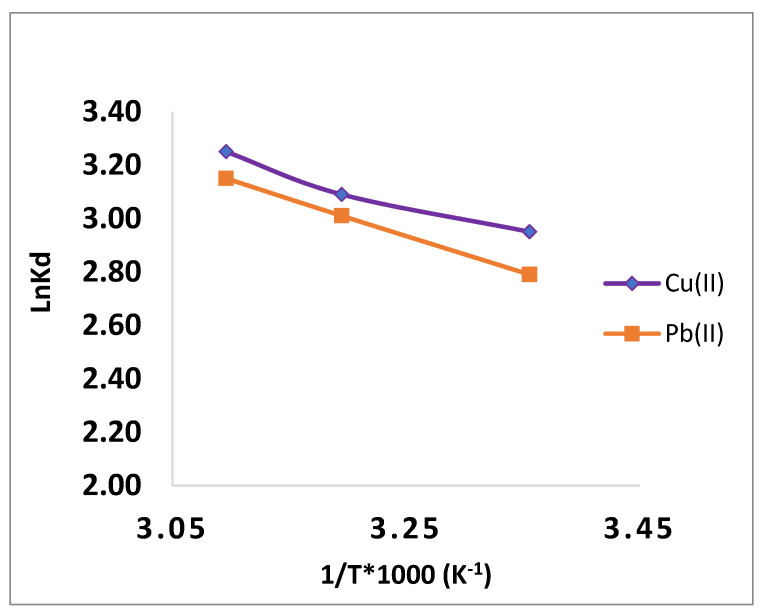
Adsorption thermodynamics of Pb(II) and Cu(II) ions onto CMOISW.

The value of ΔG^0^ (J mol^−1^) was obtained using Equation (11). The figure shows the mapping of ln Ks vs. 1/T. Table 3 lists the different thermodynamic parameters that were calculated using the slopes and crossings (Figure 10) [38].

The results for ΔS^0^ and ΔH^0^ are positive, and the adsorption process is what raises the entropy at the solid/solution contact. Additionally, the CMOISW has negative free energies, pointing to a spontaneous uptaking process in a wide range of temperatures.

Adsorption is typically used to remove metal in phases. Metal ions move from most of the solution to the outer surface of the adsorbent CMOISW in the first step, then diffuse across the boundary layer to those surfaces that initiate the metal ion adsorption at the coordination sites located at the surface of the adsorbent; at the end, intraparticle diffusion across the CMOISW particles occurs, followed by more adsorption. Further investigation was carried out using the liquid film model and the intraparticle diffusion model to achieve a thorough understanding of the adsorption mechanism.

The movement of metal ions through a liquid film enclosing the adsorbent is the longest phase of the adsorption process, according to the liquid film diffusion model described in Equation (17).

ln(1 − F) = kfd t(17)

where F is the fractional equilibrium achieved. The film diffusion coefficient is k_fd_ (min^−1^), and its formula is (F = q_t_/q_e_).

**Figure 11:**
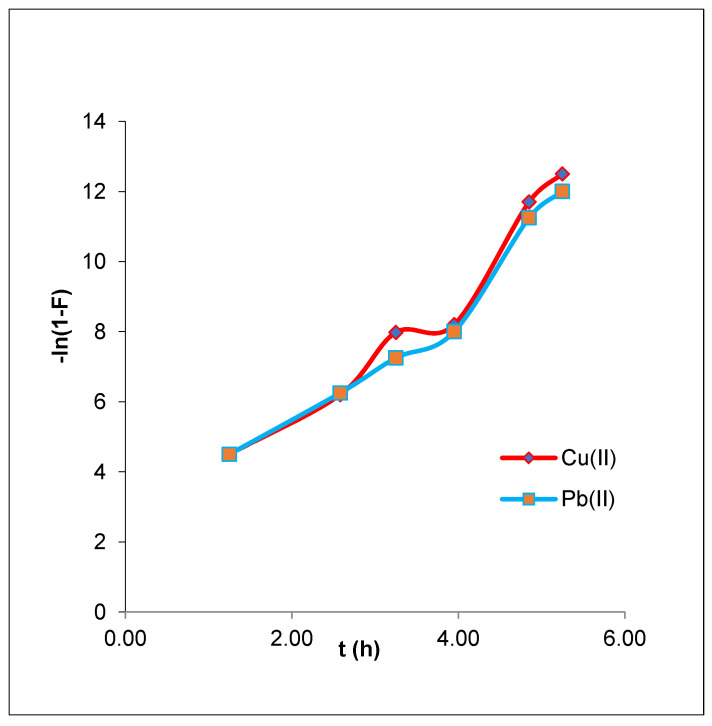
Liquid film diffusion model plots for the adsorption of CMOISW-based composites.

According to Equation (15), if plotting ln(1 − F) vs. t produced a straight line that passed through the origin, then the adsorption process includes diffusion through a liquid film around the CMOISW. Q_e_ is the equilibrium (mg/g) adsorption capacity (Figure 11).

The graph shown in Figure 11 does not exhibit linear lines crossing the origin, and has extremely low R^2^ values of 0.9036 and 0.9198 for Pb(II) and Cu(II), respectively. This suggests that the step determining velocity was not the diffusion of ions via the liquid film surrounding the CMOISW. These results suggest that, although not the slowest stage in determining the rate, the liquid film’s diffusion pattern may have an impact on the adsorption of metal ions by CMOISW especially at the beginning of adsorption, as presented in Table 3.

## 4. Conclusions

The solid waste of the olive industry was successfully converted to a material with a 3D structure for application in wastewater purification. The target 3D polymer was synthesized in a three-step process involving purification of OISW from residual olive oil and other extractives, carboxymethylation of the purified OISW by reacting it with sodium chloroacetate under alkaline conditions, and thermally curing the carboxymethylated OISW. AFM, TGA, and FT-IR techniques were used in the polymer analysis. AFM results showed that the polymer particles are nanosized. The ideal adsorption circumstances were determined. The kinetic study of the metal ions Pb(II) and Cu(II) showed that the adsorption process follows the pseudo-second-order kinetics model. Thermodynamic analysis produced negative Gibbs free energy values, suggesting that Pb(II) and Cu(II) spontaneously coordinated to the coordination site on the polymer surface. The prepared 3D polymeric material was utilized in removing the toxic metal ions from wastewater; it showed high efficiency toward most of the metal ions present in the sewage sample. The obtained results show that the 3D polymer prepared in this work from waste material could be useful in commercial applications.

## Figures and Tables

**Table 1:** Percent removal of metal ions from real sample of wastewater.

Metal Ions	Initial Concentration	Adsorption Efficiency of CMOISW	
	(ppm)		
		Final Concentration	%Removal
Ag	0.019	0.001	94%
Al	7.668	3.094	59%
As	0.624	0.360	42%
B	11.330	7.131	37%
Bi	7.176	0.527	92%
Cr	1.538	0.741	51%
Cu	14.774	0.923	93%
Fe	23.753	16.67	29%
Mo	0.515	0.192	62%
Na	3.885	1.487	61%
Ni	0.484	0.308	36%
Pb	11.805	1.550	87%
Te	0.547	0.047	91%

**Table 2:** The isotherm parameters for the adsorption of Pb(II) and Cu(II) ions by CMOISW.

Metal Ions	Pb(II)	Cu(II)
Langmuir isotherm		
Q^0^ (mg/g)	2.1584	2.0312
K_L_ (L/mg)	0.098	0.1325
R^2^	0.795	0.8735
Freundlich isotherm		
1/n	0.846	0.6095
K_F_ (L/mg)	8.6639	9.2269
R^2^	0.9543	0.995

**Table 3:** Different kinetic model parameters for the CMOISW.

Metal Ions		Pb(II)	Cu(II)
Pseudo-first-order kinetic model			
K_1_ (g/mg.min)		0.664	0.769
Q_cal_ (mg/g)		12.395	12.477
R^2^		0.916	0.903
Pseudo-second-order model for adsorption of Pb (II) and Cu (II) ions onto CMOISW			
K_2_ (g/mg.min)		0.244	0.749
Q_cal_ (mg/g)		5.170	3.717
R^2^		0.991	0.993
Parameters explain the intraparticle diffusion of Pb(II) and Cu(II) ions onto CMOISW			
K_id_		27.133	22.013
Z		6.081	12.668
R^2^		0.836	0.867
Thermodynamic parameters for the adsorption of Pb(II) and Cu(II) ions onto CMOISW			
		Cu(II)	Pb(II)
∆G° (KJ/mol)	298 K	−16.657	−18.421
	313 K	−17.496	−19.349
	323 K	−18.056	−19.967
∆H° (KJ/mol)		9.387	11.517
∆S° (J/K.mol)		55.930	61.855
Liquid film diffusion model			
K_df_		2.275	2.3671
R^2^		0.9036	0.9198

## Data Availability

Not applicable.

## References

[1] Azbar N., Bayram A., Filibeli A., Muezzinoglu A., Sengul F., Ozer A. (2004). A review of waste management options in olive oil production. Crit. Rev. Environ. Sci. Technol..

[2] Ayrilmis N., Buyuksari U. (2010). Utilization of olive mill sludge in the manufacture of fiberboard. Bioresource.

[3] Ayrilmis N., Buyuksari U. (2010). Utilization if olive mill sludge in manufacture of lignocellulosic/polypropylene composite. J. Mater. Sci..

[4] Abu-Zreig M., Al-Widyan M. (2002). Influence of olive mills solid waste on soil hydraulic properties. Commun. Soil Sci. Plant Anal..

[5] Alburquerque J.A., Gonzales J., García D., Cegarra J. (2007). Effects of a compost made from the solid by-product (‘‘alperujo’’) of the two-phase centrifugation system for olive oil extraction and cotton gin waste on growth and nutrient content of ryegrass (Lolium perenne L.). Bioresour. Technol..

[6] Sellami F., Jarboui R., Hachicha S., Medhioub K., Ammar E. (2008). Co-composting of oil exhausted olive-cake, poultry manure and industrial residues of agro-food activity for soil amendment. Bioresour. Technol..

[7] Sampedro I., Giubilei M., Cajthaml T., Federici E., Federici F., Petruccioli M., D’annibale A. (2009). Short-term impact of dry olive mill residue addition to soil on the resident Microbiota. Bioresour. Technol..

[8] Shabtay A., Hadar Y., Eitam H., Brosh A., Orlov A., Tadmor Y., Izhaki I., Kerem Z. (2009). The potential of Pleurotus-treated olive mill solid waste as cattle feed. Bioresour. Technol..

[9] Giannoutsou E.P., Meintanis C., Karagouni A.D. (2000). Identification of yeast strains isolated from a two-phase decanter system olive oil waste and investigation of their ability for its fermentation. Bioresour. Technol..

[10] Aviani I., Laor Y., Medina S., Krassnovsky A., Raviv M. (2010). Co-composting of solid and liquid olive mill wastes: Management aspects and the horticultural value of the resulting composts. Bioresour. Technol..

[11] Kalmis E., Azbar N., Yıldız H., Kalyoncu F. (2008). Feasibility of using olive mill effluent (OME) as a wetting agent during the cultivation of oyster mushroom, Pleurotus ostreatus, on wheat straw. Bioresour. Technol..

[12] Zabaniotou A., Stavropoulos G., Skoulou V. (2008). Activated carbon from olive kernels in a two-stage process: Industrial improvement. Bioresour. Technol..

[13] Abu-Ashour J., Abu Qdais H., Al Widyan M. (2010). Estimation of animal and olive solid wastes in Jordan and their potential as a supplementary energy source: An overview. Renew. Sustain. Energy Rev..

[14] Cuevas M., Sánchez S., Bravo V. (2010). Juan Francisco García, Jaime Baeza, Carolina Parra, Juanita Freer. Determination of optimal pre-treatment conditions for ethanol production from olive-pruning debris by simultaneous saccharification and fermentation. Fuel.

[15] Martinez-Garcia G., Bachmann R.T., Williams C.J., Burgoyne A., Edyvean R.G.J. (2006). Olive oil waste as a biosorbent for heavy metals. Int. Biodeterior. Biodegrad..

[16] Sampedro I., D’Annibale A., Ocampo J.A., Stazi S.R. (2006). Inmaculada García-Romera. Solid-state cultures of Fusarium oxysporum transform aromatic components of olive-mill dry residue and reduce its phytotoxicity. Bioresour. Technol..

[17] Gedon S., Fengl T., Grayson M., Eckroth D. (1993). Cellulose derivatives esters In Kirk–Othmer Encyclopedia of Chemical Technology.

[18] Heinze T., Liebert T., Rustemeyer P. (2004). Chemical characteristics of cellulose acetate In Cellulose Acetates: Properties and Applications.

[19] Majewicz T.G., Padlas T.T., Grayson M., Eckroth D. (1979). Cellulose acetate and triacetate fiber. Kirk–Othmer Encyclopedia of Chemical Technology.

[20] Serad G.H., Grayson M., Eckroth D. (1979). Cellulose derivatives, ethers In Kirk–Othmer Encyclopedia of Chemical Technology.

[21] Calihan C.D., Turball A.F. (1987). Cellulose derivatives, polymers with a future in cellulose technology research. American Chemical Society Symposium Series No. 10.

[22] Amin M.N., Shahjehan M.D. (1999). Production of cellulose acetate from jute sticks. Park J. Sci. Ind. Res..

[23] Umoren S.A., Umoudoh A.J., Akpabio U.D. (2004). Conversion of agricultural waste to cellulose derivatives. Bull. Pure Appl. Sci..

[24] Duran A., Soylak M., Tuncel S.A. (2008). Poly(vinyl pyridine-poly ethylene glycol methacrylate-ethylene glycol dimethacrylate) beads for heavy metal removal. J. Hazard. Mater..

[25] Song X., Li C., Xu R., Wang K. (2012). Molecular-Ion-Imprinted Chitosan Hydrogels for the Selective Adsorption of Silver(I) in Aqueous Solution. Ind. Eng. Chem. Res..

[26] Sikder M.T., Mihara Y., Islam M.S., Saito T., Tanaka S., Kurasaki M. (2014). Preparation and characterization of chitosan– carboxymethyl-β-cyclodextrin entrapped nanozero-valent iron composite for Cu (II) and Cr (IV) removal from wastewater. Chem. Eng. J..

[27] Allouche F.-N., Guibal E., Mameri N. (2014). Preparation of a new chitosan-based material and its application for mercury sorption. Colloids Surf. Physicochem. Eng. Asp..

[28] Liu D., Li Z., Zhu Y., Li Z., Kumar R. (2014). Recycled chitosan nanofibril as an effective Cu (II), Pb (II) and Cd (II) ionic chelating agent: Adsorption and desorption performance. Carbohydr. Polym..

[29] Kyzas G.Z., Siafaka P.I., Lambropoulou D.A., Lazaridis N.K., Bikiaris D.N. (2014). Poly (itaconic acid)-Grafted Chitosan Adsorbents with Different Cross-Linking for Pb (II) and Cd (II) Uptake. Langmuir.

[30] Heidari A., Younesi H., Mehraban Z., Heikkinen H. (2013). Selective adsorption of Pb (II), Cd (II), and Ni (II) ions from aqueous solution using chitosan–MAA nanoparticles. Int. J. Biol. Macromol..

[31] Alqadami A.A., Naushad M., ALOthman Z.A., Alsuhybani M., Algamdi M. (2020). Excellent adsorptive performance of a new nanocomposite for removal of toxic Pb (II) from aqueous environment: Adsorption mechanism and modeling analysis. J. Hazard. Mater..

[32] Sen T.K. (2017). Air, Gas, and Water Pollution Control Using Industrial and Agricultural Solid Wastes Adsorbents.

[33] Afroze S., Sen T.K. (2018). A review on heavy metal ions and dye adsorption from water by agricultural solid waste adsorbents. Water Air Soil Pollut..

[34] Demirbas A. (2008). Heavy metal adsorption onto agro-based waste materials: A review. J. Hazard. Mater..

[35] Mo J., Yang Q., Zhang N., Zhang W., Zheng Y., Zhang Z. (2018). A review on agro-industrial waste (AIW) derived adsorbents for water and wastewater treatment. J. Environ. Manage..

[36] Şenol Z.M., Gül Ü.D., Gurbanov R., Şimşek S. (2021). Optimization the removal of lead ions by fungi: Explanation of the mycosorption mechanism. J. Environ. Chem. Eng..

[37] Şenol Z.M., Gül Ü.D., Şimşek S. (2019). Assessment of Pb2+ removal capacity of lichen (Evernia prunastri): Application of adsorption kinetic, isotherm models, and thermodynamics. Environ. Sci. Pollut. Res..

[38] Jodeh S., Hamed O., Melhem A., Salghi R., Jodeh D., Azzaoui K., Benmassaoud Y., Murtada K. (2018). Magnetic nanocellulose from olive industry solid waste for the effective removal of methylene blue from wastewater. Environ. Sci. Pollut. Res..

[39] Chakraborty R., Asthana A., Singh A.K., Jain B., Susan A.B.H. (2022). Adsorption of heavy metal ions by various low-cost adsorbents: A review. Int. J. Environ. Anal. Chem..

[40] Karnitz O., Gurgel L.V.A., De Melo J.C.P., Botaro V.R., Melo T.M.S., de Freitas Gil R.P., Gil L.F. (2007). Adsorption of heavy metal ion from aqueous single metal solution by chemically modified sugarcane bagasse. Bioresour. Technol..

[41] Doan H., Lohi A., Dang A., Dang-Vu T. (2008). Removal of Zn+ 2 and Ni+ 2 by adsorption in a fixed bed of wheat straw. Process Saf. Environ. Prot..

[42] Acar F.N., Eren Z. (2006). Removal of Cu (II) ions by activated poplar sawdust (Samsun Clone) from aqueous solutions. J. Hazard. Mater..

[43] Vieira M., de Almeida Neto A., Da Silva M., Carneiro C., Melo Filho M. (2014). Adsorption of lead and copper ions from aqueous effluents on rice husk ash in a dynamic system. Braz. J. Chem. Eng..

[44] Hamed O.A., Foad Y., Hamed E.M., Al-Hajj N. (2012). Cellulose powder from olive industry solid waste. BioResources.

[45] Hamed O.A., Jodeh S., Al-Hajj N., Hamed E.M., Abo-Obeid A., Fouad Y. (2015). Cellulose acetate from biomass waste of olive industry. J. Wood Sci..

[46] Hong H.-J., Lim J.S., Hwang J.Y., Kim M., Jeong H.S., Park M.S. (2018). Carboxymethlyated cellulose nanofibrils (CMCNFs) embedded in polyurethane foam as a modular adsorbent of heavy metal ions. Carbohydr. Polym..

[47] ASTM (2005). Standard Test Methods for Sodium Carboxymethylcellulose.

[48] Kumari S., Chauhan G.S., Ahn J.H. (2016). Novel cellulose nanowhiskers-based polyurethane foam for rapid and persistent removal of methylene blue from its aqueous solutions. J. Chem. Eng..

[49] Zhao Y., Truhlar D.G. (2008). The M06 suite of density functionals for main group thermochemistry, thermochemical kinetics, noncovalent interactions, excited states, and transition elements: Two new functionals and systematic testing of four M06-class functionals and 12 other functionals. Theor. Chem. Acc..

[50] Ben Hadj Ayed M., Osmani T., Ssaoui N., Berisha A., Oujia B., Ghalla H. (2019). Structures and relative stabilities of Na+ Nen (n = 1–16) clusters via pairwise and DFT calculations. Theor. Chem. Acc..

[51] Mardirossian N., Head-Gordon M. (2017). Thirty years of density functional theory in computational chemistry: An overview and extensive assessment of 200 density functionals. Mol. Phys..

[52] Inada Y., Orita H. (2008). Efficiency of numerical basis sets for predicting the binding energies of hydrogen bonded complexes: Evidence of small basis set superposition error compared to Gaussian basis sets. J. Comput. Chem..

[53] Klamt A. (2005). COSMO-RS: From Quantum Chemistry to Fluid Phase Thermodynamics and Drug Design.

[54] Liu L., Luo X., Ding L., Luo S. (2019). Application of nanotechnology in the removal of heavy metal from water. Nanomaterials for the Removal of Pollutants and Resource Reutilization.

[55] Marczewski A.W., Seczkowska M., Deryło-Marczewska A., Blachnio M. (2016). Adsorption equilibrium and kinetics of selected phenoxyacid pesticides on activated carbon: Effect of temperature. Adsorption.

[56] Weißpflog J., Gündel A., Vehlow D., Steinbach C., Müller M., Boldt R., Schwarz S., Schwarz D. (2020). Solubility and selectivity effects of the anion on the adsorption of different heavy metal ions onto chitosan. Molecules.

[57] Mészáros, Varga I., Gilányi T. (2004). Adsorption of poly (ethyleneimine) on silica surfaces: Effect of pH on the reversibility of adsorption. Langmuir.

[58] Hamed O., Abu Lail B., Deghles A., Qasem B., Azzaoui K., Abu Obied A., Algarra M., Jodeh S. (2019). Synthesis of a cross-linked cellulose-based amine polymer and its application in wastewater purification. Environ. Sci. Pollut. Res. Vol..

